# Comparative Efficacy of Anti-asthma Therapy in Non-asthmatic Cough: A Cross-Sectional Study in Dubai, United Arab Emirates

**DOI:** 10.7759/cureus.47377

**Published:** 2023-10-20

**Authors:** Syed Arshad Husain, Muhammad Omar Larik, Maryam Urooj, Muhammad Amir Javed, Jean Mary John

**Affiliations:** 1 Department of Pulmonology, King's College Hospital London, Dubai, ARE; 2 Department of Medicine, Dow International Medical College, Karachi, PAK

**Keywords:** tiotropium, ipratropium, montelukast, budesonide, inhaler, chronic cough, acute cough, cough

## Abstract

Background

Cough is one of the most common presenting complaints for physicians across the world, with the potential to result in a significant influence on one’s daily life. It is typically categorized into acute cough (<3 weeks), subacute cough (three to eight weeks), and chronic cough (>8 weeks). The lack of specific treatment guidelines and evidence-based recommendations for resolving cough creates reasonable controversy in the medical field. This retrospective study aims to identify the clinical features of cough and evaluate the comparative efficacy between different anti-asthmatic treatment modalities in the urban city of Dubai, United Arab Emirates.

Methods

A retrospective cross-sectional study was performed on patients presenting to pulmonology or respiratory outpatient clinics with complaints of cough in the absence of any known history of chronic respiratory illness (e.g., asthma). Analysis was conducted via chi-squared and analysis of variance (ANOVA) testing.

Results

A total of 308 patients were eligible for inclusion, with 273 patients presenting for follow-up. Overall, patients with acute, subacute, and chronic coughs had similar clinical presentations, with no statistically significant differences noted. However, patients with pets were more likely to develop an acute cough (p = 0.04). Moreover, the follow-up outcomes of acute, subacute, and chronic cough were similar, with no significant statistical difference noted. Furthermore, patients receiving dual therapy using budesonide and montelukast, and patients receiving triple therapy using budesonide, montelukast, and tiotropium/ipratropium were most likely to gain complete relief of their symptoms, although triple therapy treatment was also associated with the highest rate of null improvement (p = 0.012). Additionally, chronic cough patients were more likely to be subject to higher C-reactive protein (CRP) levels in comparison to other cohorts (p = 0.26).

Conclusion

The comparative superiority of dual therapy using budesonide and montelukast, and triple therapy using budesonide, montelukast, and tiotropium/ipratropium were highlighted in this study. In the sparseness of specific treatment guidelines and evidence-based recommendations for cough, the use of anti-asthmatic therapy for cough patients has shown favorable results. Moreover, the lack of clinical differences between acute, subacute, and chronic cough may result in difficulties with the treatment of cough patients. To arrive at a valid conclusion, further comprehensive studies with larger and more diversified sample populations are encouraged.

## Introduction

Cough is one of the most common complaints presented by primary care physicians across the world, with most cases of severely acute or persistently chronic cough being referred to respiratory clinics for further inquiry [1,2]. Up to 40% of adults presenting to specialist respiratory clinics have an unknown etiology behind them, and are usually refractory to standard lines of care, creating an immensely conflicting array of treatment opportunities between various physicians [3]. There may be various pathophysiological mechanisms at play with respect to cough, ranging from low levels of chemical, mechanical, and thermal exposure to hypersensitivity reactions [4]. It is often known to be an extremely burdensome symptom for the patient, greatly affecting one’s quality of life and daily routine of activities, resulting in sleep disturbance, disordered breathing, psychological issues including anxiety and depression, social issues including isolation, and limited sports or recreational activities [5-7]. Furthermore, an increased economic burden has also been observed in such patients, leading to a subsequent lower quality of life [8].

The treatment of cough is thought to be more complex than it initially seems. Traditionally, cough may be classified as acute cough (<3 weeks), subacute cough (three to eight weeks), or chronic cough (>8 weeks) [9]. Cough patients, especially those with an acute nature, may be generally prescribed antitussive drugs (cough syrups) with doubtful and largely unproven clinical efficacy, as proved by a meta-analysis of 15 trials conducted by Schroeder et al. [10]. Likewise, antihistamines were not observed to be any more beneficial than the placebo. Thus, the potential advantage of using asthma-like treatment in such patients, with no known history of asthma, may prove to be therapeutic. Moreover, there has been extensive research conducted in the pediatric field, assessing the treatment outcomes of various anti-asthma therapies, with inhalers demonstrating a potential benefit in cough patients [11].

Therefore, in light of the rising number of refractory coughs in the absence of any known asthma diagnosis and the sparseness of existing research, this retrospective study was conducted in order to compare the efficacy of inhalers for acute, subacute, and chronic cough, in a racially and ethnically diverse setting in the city of Dubai, United Arab Emirates, featuring a majority expatriate population. These indiscriminate results will be highly generalizable to the Asian and Middle Eastern populations in other regions across the world and will aid in the development of alternative treatment strategies involving anti-asthma therapy, as opposed to cough syrups or antihistamines.

## Materials and methods

Study design

This retrospective cross-sectional analysis was conducted at King’s College Hospital London, Dubai, United Arab Emirates, among patients visiting the pulmonology or respiratory outpatient clinics from January 2022 to July 2023, presenting with complaints of cough in the absence of known chronic respiratory illness. The local ethical approval was obtained by the Research and Ethics Committee of King’s College Hospital London, Dubai, United Arab Emirates (KCH/MOI/740). Follow-up was defined as the patient's subsequent presentation to the pulmonology or respiratory outpatient clinic within the span of seven to 21 days from the initial consultation.

Data collection and extraction

The electronic medical record system of the hospital was utilized for data collection. The following data was extracted for the purpose of this study: (i) age, (ii) gender, (iii) BMI, (iv) history of smoking, (v) history of pets, (vi) lab parameters, (vii) spirometry results, and (viii) x-ray results highlighting chest abnormalities related to cough. The following laboratory parameters were included (i) fractionated exhaled nitric oxide (FeNO), (ii) serum immunoglobulin E (IgE), (iii) absolute eosinophil count, and (iv) C-reactive protein (CRP). The following symptomatic complaints of the respiratory system were included (i) cough, (ii) cough with sputum, (iii) wheeze, (iv) shortness of breath, and (v) fever. One of three treatment strategies was prescribed to the patient, including monotherapy using inhalers (budesonide), dual therapy using inhalers and anti-leukotrienes (budesonide and montelukast), and triple therapy using inhalers, anti-leukotrienes, and anticholinergics (budesonide, montelukast, and tiotropium/ipratropium). The effectivity of response to therapy was evaluated based on the relief of the cough symptom, as reported by patients on follow-up visits.

Classification of cough

For the purpose of this study, the patients were assigned into one of three cohorts, including (i) acute cough defined as a cough for <3 weeks, (ii) subacute cough defined as a cough for three to eight weeks, and (iii) chronic cough defined as a cough for >8 weeks. This stratification was defined on the basis of international and established guidelines [9].

Inclusion and exclusion criteria

The following patient characteristics warranted inclusion into our retrospective study: (i) age equal to or above 18, (ii) presenting with a complaint of cough, (iii) prescribed one of the three treatments under consideration in this study, and (iv) absence of any known chronic respiratory illnesses, such as asthma, bronchiectasis, and chronic obstructive pulmonary disease (COPD). Any patients below the age of 18, or with a medical history of chronic respiratory disease, were excluded from the inclusion process.

Statistical analyses

All statistical analyses were conducted through the “Statistical Package for Social Sciences” (SPSS) version 20.0 (IBM Corp., Armonk, NY). Multiple comparisons were carried out using the chi-squared test to evaluate the association between the categorical variables in the study, and analysis of variance (ANOVA) testing was conducted for continuous numerical datasets. In all comparisons, a p-value < 0.05 was considered statistically significant.

## Results

Demographic characteristics of the study population

In this retrospective study, there were 308 patients eligible for inclusion throughout the study period. A total of 150 patients presented with acute cough, 57 patients presented with subacute cough, and 101 patients presented with chronic cough. The overall median age of the included study population was 40.0 years (IQR: 33.0-49.0), with no significant differences between each cohort (p = 0.64). There was a total of 158 male participants and 150 female participants, with no significant differences between each cohort (p = 0.23). The mean BMI of the included study population was 26.7 kg/m^2^ (SD: 6.0), with no significant differences between each cohort (p = 0.79). The majority of the included study population were non-smokers (n = 210), followed by regular smokers (n = 68). The smoking status was not significantly different among the assigned cohorts. Most participants were non-pet owners (n = 243); however, there were significant differences observed between pet ownership and the type of cough (p = 0.03), where pet owners were more likely to suffer from acute cough. The demographic characteristics of the included study population are available in Table 1.

**Table 1:** Patient baseline characteristics of included study population. n: Number of Participants, y: Years, IQR: Interquartile Range, BMI: Body Mass Index, SD: Standard Deviation, FeNO: Fractional Exhaled Nitric Oxide, IgE: Immunoglobulin E, CRP: C-reactive Protein.

Variable		Overall (n = 308)	Acute Cough (n = 150)	Subacute Cough (n = 57)	Chronic Cough (n = 101)	P-value
Median Age, y (IQR)		40.0 (33.0-49.0)	40.0 (33.0-47.0)	42.0 (33.5-53)	39.0 (33.0-49.0)	0.64
Mean BMI (SD)		26.7 (6.02)	26.5 (4.83)	27.09 (5.94)	26.88 (7.52)	0.79
Gender	Male, n (%)	158 (51.3)	84 (56.0)	20 (35.1)	54 (53.5)	0.23
	Female, n (%)	150 (48.7)	66 (44.0)	37 (64.9)	47 (46.5)	
Smoking History	Non-smoker, n (%)	210 (68.2)	102 (68.0)	37 (64.9)	71 (70.3)	0.49
	Current smoker, n (%)	68 (22.1)	37 (24.7)	12 (21.5)	19 (18.8)	
	Former smoker, n (%)	23 (7.5)	7 (4.7)	6 (10.5)	10 (9.9)	
	Vape/e-cigarette, n (%)	7 (2.3)	4 (2.7)	2 (3.5)	1 (1.0)	
Pet History	Yes, n (%)	65 (21.1)	38 (25.3)	6 (10.5)	17 (16.8)	0.038
	No, n (%)	243 (78.9)	112 (74.7)	51 (89.5)	84 (83.2)	
Lab Parameters	FeNO, ppb (SD)	41.90 (41.78)	38.86 (34.58)	35.63 (33.79)	50.03 (52.96)	0.10
	IgE, IU/mL (SD)	722.21 (3286.38)	389.25 (490.61)	748.82 (3205.84)	1169.15 (5076.55)	0.31
	Eosinophil, x10^9^ (SD)	0.43 (1.57)	0.45 (1.77)	0.22 (0.26)	0.52 (1.73)	0.59
	CRP, mg/dL (SD)	13.47 (30.20)	6.50 (8.20)	6.63 (7.79)	26.49 (48.01)	0.026
Spirometry	Normal, n (%)	80 (26.0)	29 (19.3)	20 (35.1)	31 (30.7)	0.18
	Obstructive, n (%)	99 (32.1)	53 (35.3)	20 (35.1)	26 (25.7)	
	Restrictive, n (%)	27 (8.8)	14 (9.3)	4 (7.0)	9 (8.9)	
	Unreported, n (%)	102 (33.1)	54 (36.0)	13 (22.8)	35 (34.7)	
X-ray Status	Normal, n (%)	161 (52.3)	63 (42.0)	40 (70.2)	58 (57.4)	0.080
	Abnormal, n (%)	39 (12.7)	23 (15.3)	5 (8.8)	12 (11.9)	
	Unreported, n (%)	108 (35.0)	64 (42.7)	12 (21.0)	31 (30.7)	

Comparison of clinical features and investigative findings stratified by acute, subacute, and chronic cough

The following investigative parameters were compared between participants with acute, subacute, or chronic cough, including (i) FeNO levels, (ii) serum IgE, (iii) eosinophil counts, and (iv) CRP. The chronic cough cohort was observed to have the highest FeNO (mean = 50.0 ppb), serum IgE (mean = 1,169 IU/mL), and eosinophil counts (mean = 0.52 x 10^9^), although these differences failed to reach statistical significance. However, the CRP levels (mean = 26.49 mg/dL) showed a statistically significant elevation in the chronic cough group (p = 0.026), with similar mean values observed for the acute and subacute cough cohorts. 

All patients in acute, subacute, and chronic cough cohorts were most likely to be associated with sputum secretion and wheezing. The symptoms of throat pain and fever were comparatively less likely across all the included cohorts. Overall, there were no statistically significant differences between the symptomatic presentation of each cohort of the included study population (p = 0.58).

A total of 273 patients presented for follow-up. Subacute cough sufferers were more likely to suffer from a lack of improvement on follow-up and were also least likely to receive complete relief of their symptoms on follow-up. However, there were no statistically significant differences between the three included cohorts (p = 0.20). Data comparing clinical features and follow-up improvement of cough, stratified by type of cough, is available in Table 2.

**Table 2:** Comparison of clinical features and follow-up improvement of cough stratified by acute, subacute, and chronic cough. n: Number of Participants, MT: Mono-therapy, DT: Dual Therapy, TT: Triple Therapy.

Variable		Overall (n = 308)	Acute Cough (n = 150)	Subacute Cough (n = 57)	Chronic Cough (n = 101)	P-value
Presenting Complaints	Cough, n (%)	308 (100.0)	150 (100.0)	57 (100.0)	101 (100.0)	0.58
	Cough with Sputum, n (%)	65 (21.1)	32 (21.3)	10 (17.5)	23 (22.8)	
	Wheeze, n (%)	71 (23.1)	33 (22.0)	16 (28.1)	22 (21.8)	
	Throat, n (%)	28 (9.1)	16 (10.7)	4 (7.0)	8 (7.9)	
	Fever, n (%)	11 (3.6)	7 (4.7)	3 (5.3)	1 (1.0)	
Treatment	MT, n (%)	122 (39.6)	71 (47.3)	18 (31.5)	33 (32.7)	<0.00001
	DT, n (%)	133 (43.2)	60 (40.0)	14 (24.6)	59 (58.4)	
	TT, n (%)	53 (17.2)	19 (12.7)	25 (43.9)	9 (8.9)	
Follow-up of Cough	No improvement, n (%)	57 (20.9)	28 (21.6)	16 (29.6)	13 (14.3)	0.20
	Slight improvement, n (%)	90 (33.0)	38 (30.0)	17 (31.5)	35 (38.5)	
	Complete improvement, n (%)	126 (46.1)	62 (48.4)	21 (38.9)	43 (47.2)	

Comparison of outcomes stratified by treatment

The following treatment regimens were utilized by the study population: (i) monotherapy using budesonide only, (ii) dual therapy using budesonide and montelukast, and (iii) triple therapy using budesonide, montelukast, and tiotropium/ipratropium. A total of 273 patients presented for follow-up. Acute cough patients were more likely to be prescribed a monotherapy regimen, subacute cough patients were more likely to be prescribed a triple therapy regimen, and chronic cough patients were more likely to be prescribed a dual therapy regimen. Overall, there were statistically significant differences between the prescription habits through the three cohorts (p < 0.00001). Patients undergoing dual therapy and triple therapy had a comparatively higher likelihood of presenting with complete relief of their symptoms on follow-up, whereas patients undergoing monotherapy were comparatively less likely to receive complete relief of their symptoms on follow-up. In contrast, patients undergoing triple therapy were comparatively more likely to have no relief on follow-up. Ultimately, there were statistically significant differences within the follow-up outcomes between each treatment cohort (p = 0.012). Data comparing treatment outcomes of cough, stratified by type of treatment used, are available in Table 3.

**Table 3:** Comparison of baseline characteristics and follow-up improvement of cough stratified by treatment used. n: Number of Participants, y: Years, IQR: Interquartile Range, BMI: Body Mass Index, SD: Standard Deviation, FeNO: Fractional Exhaled Nitric Oxide, IgE: Immunoglobulin E, CRP: C-reactive Protein.

Variables		Overall (n = 308)	MT (n = 122)	DT (n = 133)	TT (n = 53)	P-value
Median Age, y (IQR)		40.0 (33.0-49.0)	41.0 (35.0-49.0)	38.0 (31.0-47.0)	41.0 (34.0-51.5)	0.071
Mean BMI (SD)		26.7 (6.02)	25.9 (4.63)	27.1 (7.00)	27.6 (6.08)	0.14
Gender	Male, n (%)	158 (51.3)	63 (51.6)	73 (54.9)	22 (41.5)	0.26
	Female, n (%)	150 (48.7)	59 (48.4)	60 (45.1)	31 (58.5)	
Smoking History	Non-smoker, n (%)	210 (68.2)	86 (70.5)	87 (65.4)	37 (69.8)	0.92
	Current smoker, n (%)	68 (22.1)	26 (21.3)	31 (23.3)	11 (20.8)	
	Former smoker, n (%)	23 (7.5)	8 (6.6)	12 (9.0)	3 (5.7)	
	Vape/e-cigarette, n (%)	7 (2.3)	2 (1.6)	3 (2.3)	2 (3.7)	
Pet History	Yes, n (%)	65 (21.1)	25 (20.5)	32 (24.1)	8 (15.1)	0.39
	No, n (%)	243 (78.9)	97 (79.5)	101 (75.9)	45 (84.9)	
Lab Parameters	FeNO, ppb (SD)	41.90 (41.78)	38.17 (35.29)	46.01 (47.19)	40.25 (40.75)	0.42
	IgE, IU/mL (SD)	722.21 (3286.38)	329.28 (424.90)	546.27 (845.39)	919.35 (3289.56)	0.14
	Eosinophil, x10^9^ (SD)	0.43 (1.57)	0.39 (1.41)	0.53 (1.93)	0.21 (0.28)	0.57
	CRP, mg/dL (SD)	13.47 (30.20)	18.50 (40.64)	10.07 (20.46)	11.89 (26.93)	0.58
Spirometry	Normal, n (%)	80 (26.0)	32 (26.2)	36 (27.1)	12 (22.6)	0.53
	Obstructive, n (%)	99 (32.1)	30 (24.6)	46 (34.6)	23 (43.4)	
	Restrictive, n (%)	27 (8.8)	8 (6.6)	14 (10.5)	5 (9.4)	
	Unreported, n (%)	102 (33.1)	52 (42.6)	37 (27.8)	13 (24.6)	
X-ray Status	Normal, n (%)	161 (52.3)	50 (41.0)	79 (59.4)	32 (60.4)	0.33
	Abnormal, n (%)	39 (12.7)	17 (13.9)	16 (12.0)	6 (11.3)	
	Unreported, n (%)	108 (35.0)	55 (45.1)	38 (28.6)	15 (28.3)	
Type of Cough	Acute, n (%)	150 (48.7)	71 (58.2)	60 (45.1)	19 (35.8)	<0.00001
	Subacute, n (%)	57 (18.5)	18 (14.8)	14 (10.5)	25 (47.2)	
	Chronic, n (%)	101 (32.8)	33 (27.0)	59 (44.4)	9 (17.0)	
Follow-up of Cough	No improvement, n (%)	57 (20.9)	20 (19.6)	24 (19.7)	13 (26.5)	0.012
	Slight improvement, n (%)	90 (33.0)	45 (44.1)	36 (29.5)	9 (18.4)	
	Complete improvement, n (%)	126 (46.1)	36 (35.3)	62 (50.8)	28 (57.1)	

## Discussion

This retrospective analysis of patients with no known history of asthma highlighted the similarity between the symptomatic presentation and follow-up results of acute, subacute, and chronic cough cohorts. The symptoms of cough were commonly associated with the symptoms of sputum release and wheezing. However, patients receiving both budesonide and montelukast, and patients receiving budesonide, montelukast, and tiotropium were most likely to receive benefits and be subject to improved rates of cure on follow-up. With the exceedingly foreign and largely expatriate population of the city of Dubai in the United Arab Emirates, these results aim to provide a generalizable understanding of using anti-asthmatic therapy in non-asthmatic patients in an urban, metropolitan city.

In a study conducted by Yi et al., it was observed that the efficacy of montelukast alone, budesonide alone, or a combination of both were efficacious in overcoming the symptom of cough, but also decreased cough reflex sensitivity, improving airway inflammation, and providing an overall antitussive effect [12]. This largely remains consistent with our results, where nearly 80% of patients reported some form of relief of their cough symptomatology; although the combination of dual therapy using budesonide and montelukast was seen as a superior choice for our setting, reporting approximately 15% increase in the complete eradication of cough in comparison to monotherapy using budesonide only. Additionally, the combination of inhaled corticosteroids and montelukast was proven to be effective for relieving cough, in comparison to inhaled corticosteroids alone, in a large-scale meta-analysis of fifteen randomized controlled trials [13]. In contrast, a randomized controlled trial evaluating the efficacy of anticholinergics (i.e., tiotropium) in relieving acute cough demonstrated a significant decrease in cough reflex sensitivity in patients, although the antitussive action of anticholinergics is thought to be through mechanisms other than the traditional bronchodilatory approach [14]. Similarly, the results of this study remain consistent with our results, where triple therapy using budesonide, montelukast, and tiotropium demonstrated the highest efficacy in relieving symptomatic cough. Despite the triple therapy combination treatment illustrating the greatest efficacy in resolving cough, it also had the highest rate of null improvement within our study. This unusual and unexpected finding may be due to the smaller sample size (n = 53) of this cohort, indicating the need for further large-scale studies.

In our analysis, it was revealed that chronic cough exhibited significantly increased levels of CRP in comparison to other cohorts. In a study conducted by Melbye et al., it was observed that viral upper respiratory infection resulted in a moderately elevated CRP level (10-60 mg/L), indicating its usefulness in its prediction for the disease, especially in acute cough patients [15]. However, this observation did not apply to bacterial upper respiratory infections. These results go against our study findings; however, it is important to note our limited sample size and differences in study design and methodology. Additionally, it has also been revealed that elevated serum biomarkers have been associated with asthma; however, CRP failed to reveal a statistically significant association [16]. Ultimately, our results may be attributed to long-standing inflammatory processes involving the respiratory system, causing chronically elevated levels of CRP in the chronic cough cohort. Alternatively, the smaller and limited sample size, in conjunction with the single-centered retrospective collection of data may induce heterogeneity within the results, leading to such findings.

There are several clinical implications that arise from the results of our cross-sectional analysis. Firstly, we observed a similarity in cough improvement rates between the three cohorts of acute, subacute, and chronic cough. The current recommendation for treating acute cough involves the use of first-generation antihistamines with decongestants [17]. However, the use of anti-asthmatic therapy may prove useful in treating persistent or refractory patients with a particularly aggressive acute cough. On the other hand, the gray area of treating subacute cough suffers from conflicting guidelines and a lack of evidence-based recommendations for treatment. However, subacute cough is typically associated with a non-progressive and self-limiting nature, typically resolving on its own. Therefore, the risk of overtreatment arises in such cases, where a meta-analysis conducted by Speich et al. failed to reveal any effective treatment modality for subacute cases, highlighting the weak evidence and the need for future studies [18]. In refractory cases, where treatment of the underlying cause yields no improvement, then neuromodulatory treatment including low-dose opioids, gabapentin, or speech-language therapy may be a suitable consideration [19]. Although there are no specific treatment modalities for chronic cough, the new class of drugs (purinergic P2X3 receptors) are under experimental testing, potentially serving as the first-line therapeutic modality in the foreseeable future [19].

This study includes various limitations that must be highlighted. Firstly, the retrospective nature of data collection has been associated with the risk of missing or potentially incorrect information, while also being affected by selection and recall bias, ultimately affecting the validity and generalizability of our results [20]. Secondly, there were certain patients lost to follow-up, possibly indicating symptomatic improvement and a lack of need for re-evaluation, resulting in the skewing of our analysis. Third, the limited timeframe of the retrospective data collection relies on the assumption that these trends will not change and remain generalizable. Thirdly, the sample population included residents and citizens of an urban metropolitan city, and hence, may not be applicable to other regions of the world, especially those in rural regions. Fourthly, the patient inclusion was performed on the basis of past medical history, which may prove unreliable in cases of missing or inaccurate electronic records. In particular, the chronic cough cohort may marginally overlap with unknown asthmatic patients, potentially skewing our results. Finally, the assumption of absolute compliance was made with respect to the patient’s treatment strategies, hence introducing considerable bias in patients with poor compliance with their treatment regimen. Ultimately, this highlights the need for future robust and randomized studies in order to validate our results.

## Conclusions

This retrospective study revealed statistically similar rates of outcomes on follow-up between acute, subacute, and chronic cough cohorts. In contrast, dual therapy using budesonide and montelukast, and triple therapy using budesonide, montelukast, and tiotropium/ipratropium were comparatively associated with statistically superior rates of symptomatic relief on follow-up. This highlights the potential efficaciousness of anti-asthmatic therapy for cough patients, in light of weak evidence-based studies, cough-specific targeted therapy, and treatment recommendations available for the treatment of cough. In order to arrive at a valid conclusion, further comprehensive studies, with larger and more diversified sample populations, are encouraged.
